# Hemophagocytic Lymphohistiocytosis with Predominant T-Lymphocytes in Young Child: An Unusual Presentation of Evolving Acute Myeloid Leukemia

**DOI:** 10.3390/jcm14051511

**Published:** 2025-02-24

**Authors:** Aida I. Richardson, Kai Lee Yap, Katrin Leuer, Shunyou Gong

**Affiliations:** Department of Pathology & Laboratory Medicine, Ann & Robert H. Lurie Children’s Hospital of Chicago, Northwestern University Feinberg School of Medicine, Chicago, IL 60611, USA; klyap@luriechildrens.org (K.L.Y.); kmcarlson@luriechildrens.org (K.L.); sgong@luriechildrens.org (S.G.)

**Keywords:** malignancy-associated hemophagocytic lymphohistiocytosis, acute myeloid leukemia, *KMT2A::MLLT3*

## Abstract

**Background:** Hemophagocytic lymphohistiocytosis (HLH) is an aggressive, life-threatening condition commonly observed in young children. Distinguishing primary HLH from secondary HLH, such as malignancy-associated HLH, can be challenging, potentially leading to misdiagnosis and inappropriate treatment. **Case presentation:** A 16-month-old female presented with fever, decreased appetite, and rhinorrhea. A review of the peripheral blood smear revealed anemia and leukopenia, with absolute neutropenia characterized by a high lymphocyte count (approximately 80% were T cells by flow cytometry). Flow cytometry was negative for immunophenotypically abnormal cells. Initially, the cytopenia was attributed to a viral infection. However, the cytopenia did not improve, and a bone marrow evaluation revealed evidence of HLH but no immunophenotypically abnormal population. An extensive work-up for HLH, including next-generation sequencing (NGS) and cytogenetic testing identified the *KMT2A::MLLT3* fusion transcript, indicating malignancy-associated HLH in the setting of evolving leukemia. Because there was no increase in blasts or immunophenotypically abnormal cells, the diagnosis of leukemia could not be made at that time. The patient was closely monitored and, seven weeks later, was diagnosed with acute myeloid leukemia/acute monocytic leukemia. In addition to the *KMT2A::MLLT3* fusion, pathogenic variants in the *PTPN11* and *FLT3* genes were detected by NGS. **Conclusions:** The presentation of evolving acute monocytic leukemia can be nonspecific, mimicking conditions such as HLH, without an initial increase in immature cells or monocytes. Maintaining a broad differential diagnosis and including comprehensive molecular genetic testing may facilitate early diagnosis and appropriate treatment.

## 1. Introduction

Hemophagocytic lymphohistiocytosis (HLH) is a relatively rare, aggressive, and life-threatening syndrome characterized by excessive inflammation and tissue destruction due to abnormal immune activation, leading to multi-organ failure and death, making prompt and appropriate treatment imperative [1,2,3]. The hyperinflammatory/dysregulated immune state is thought to be caused by the absence of normal downregulation by activated macrophages and lymphocytes.

HLH is classified into primary (genetic) and secondary (acquired) forms. While HLH can occur at any age, primary HLH, most commonly familial HLH, typically affects infants and young children and is caused by inherited defects in natural killer cells and cytotoxic T cells function, often involving the perforin–granzyme cell-death pathway [3,4,5,6]. Perforin creates pores in the target cell membrane, allowing granzymes to enter and induce apoptosis. In primary HLH, defects in these proteins impair the ability of cytotoxic cells to eliminate infected or abnormal cells, leading to the uncontrolled immune response. Secondary HLH is more common in adults and is thought to have a multifactorial cause, with one underlying cause often being predominant [3]. It is often triggered by infections, malignancy, or autoimmune diseases.

Malignancy-associated HLH, a type of secondary HLH, is uncommon in children, representing only 1.9% to 8% of pediatric HLH cases [7,8,9]. In contrast with primary HLH, malignancy-associated HLH is particularly rare in infants and young children, typically occurring in older children with a median age of 7 to 12 years [7,8,9]. Nonetheless, it should be noted that malignancy-associated HLH not only occurs at the time of presentation of malignancy but also in the setting of therapy-induced immunosuppression in patients who have already achieved remission [10,11]. Based on this, malignancy-associated HLH has been further classified into malignancy-triggered HLH (HLH at the onset of malignancy) and HLH occurring during chemotherapy (Ch-HLH) [8,9]. Despite the conflicting data on which of the two subtypes (malignancy-triggered or Ch-HLH) is more common in children, all studies agree that lymphoid malignancies account for the majority of malignancy-triggered HLH [7,8,9]. Notably, myeloid malignancies are rare and primarily observed in setting of intensive chemotherapy or post-hematopoietic stem-cell transplantation for acute myeloid leukemia, with infections being the most frequent trigger [11,12,13].

Here, we report a diagnostically challenging case of malignancy-triggered HLH in a setting of evolving acute myeloid leukemia, initially presenting as HLH with an abundance of T lymphocytes.

## 2. Materials and Methods

### 2.1. Histopathologic Analysis

Bone marrow biopsy specimens were decalcified, fixed in formalin, and embedded in paraffin to prepare tissue blocks, which were then sectioned and stained. Immunohistochemical analysis on bone marrow biopsies included the antibodies ALK-1 (Leica Biosystems, Buffalo Grove, IL, USA), CD30 (Leica Biosystems, Buffalo Grove, IL, USA), and CD1a (Cell Marque, Rocklin, CA, USA), while Epstein–Barr virus (EBV)-encoded RNA (EBER; Leica Biosystems, Buffalo Grove, IL, USA) was used to detect EBV-infected cells. Staining was performed with an automated Bond Prime slide stainer (Leica). The reticulin special stain (Poly Scientific, Bay Shore, NY, USA) was used to evaluate bone marrow fibrosis.

### 2.2. Flow Cytometry Analysis

Diagnostic flow cytometry was performed on peripheral blood and/or bone marrow samples collected in heparinized tubes. Samples were analyzed within 24 h of collection. An immunophenotypic analysis was performed in all cases based on a predefined diagnostic panel of antibodies. The diagnostic samples were tested with the following antibodies: CD2, cytoplasmic CD3, CD7, CD58 (all from BD Pharmingen, San Diego, CA, USA), surface CD3, CD4, CD5, CD10, CD13, CD14, CD22, CD24, CD25, CD34, CD36, CD38 (all from BD Biosciences, San Jose, CA, USA), CD8, CD15, CD19, CD33, CD117 (all from Beckman Coulter, Miami, FL, USA), CD20 (BioLegend, San Diego, CA, USA), CD45 (Invitrogen, Carlsbad, CA, USA), CD56, HLA-DR (both from BD Horizon, San Jose, CA, USA), CD79a, cytoplasmic myeloperoxidase (both from Daco, Carpinteria, CA, USA), and terminal deoxynucleotidyl transferase (TdT; SuperTech, Bethesda, MD, USA). For detection of cytoplasmic/nuclear antigens, BD FACS Permeabilizing Solution 2 was used. Immunophenotypic flow cytometric analysis was performed on the flow cytometer (Becton Dickinson FACS Canto II) and analyzed with FCS Express. Results were obtained by gating the singlet cells with side scatter (SSC) versus forward scatter (FSC), followed by SSC versus CD45.

Samples for minimal residual disease (MRD) flow cytometry at the end of induction and follow-up were performed on bone marrow samples that were sent to a reference lab.

### 2.3. Conventional Cytogenetic Analysis and Interphase Fluorescence In Situ Hybridization (FISH)

Cytogenetic testing, either by conventional G-banding chromosome analysis or fluorescence in situ hybridization (FISH), was performed on fresh bone marrow samples. The cytogenetic karyotypes were reported according to the International System for Human Cytogenetic Nomenclature. FISH was performed with the following probe set: KMT2A (MLL) dual-color break apart (Abbott Molecular, Des Plaines, IL, USA). A total of 200 interphase nuclei were scored per probe set by two individuals. The cutoff value for a positive result was >1.5%.

### 2.4. Next-Generation Sequencing (NGS)

Next-generation sequencing (NGS) was performed on RNA and DNA isolated from fresh bone marrow aspirate. RNA samples were reverse transcribed to cDNA. Samples of cDNA and DNA were target-amplified using the Ion AmpliSeq Library Kit (ThermoFisher: Mississauga, ON, Canada) in conjunction with the Oncomine Childhood Cancer Research Assay. Sequencing was performed on the Ion Torrent S5 (ThermoFisher: Waltham, MA, USA) sequencer and analyzed using Ion Reporter and a customized bioinformatics pipeline to identify clinically significant genetic variants and RNA fusion transcripts. Clinically significant results were reported according to CAP/AMP/ASCO guidelines.

## 3. Clinical Case

### 3.1. Initial Presentation

**Figure 1:**
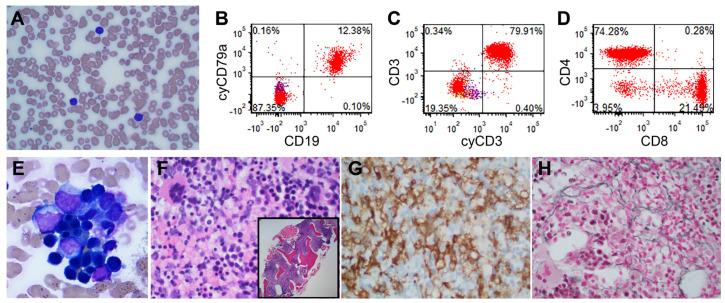
Peripheral blood and bone marrow at presentation. (**A**) Peripheral blood with anemia and leukopenia, showing mostly lymphocytes (Wright–Giemsa stain, magnification ×400). (**B**) B cells comprise about 12% of the white blood cells. (**C**) Approximately 80% of white blood cells are T cells. (**D**) The CD4 to CD8 ratio is approximately 3.5:1 by flow cytometry. (**E**) Bone marrow aspirates with lymphocytes and some histiocytes showing evidence of hemophagocytosis (Wright–Giemsa stain, magnification ×1000). (**F**) Hypercellular bone marrow core biopsy (inset magnification ×40) with mostly lymphocytes and histiocytes (Hematoxylin and Eosin stain, magnification ×400). (**G**) CD163 stain highlighting histiocytes (magnification ×400). (**H**) Reticulin stain shows mild increase in fibrosis (MF-1) (magnification ×400).

A 16-month-old female presented at an outside hospital with fever, decreased appetite, rhinorrhea, and fatigue for 8 days. Laboratory studies performed at the outside hospital revealed neutropenia and anemia requiring a blood transfusion due to reported hemoglobin levels of 6.9 g/dL. Upon admission to our hospital, her peripheral blood showed anemia (hemoglobin 7.8 g/dL), leukopenia (WBC 3.3 K/μL), and a platelet count within normal limits (156 K/μL). The white blood cell differential counts revealed absolute neutropenia (neutrophils 0.4 K/uL) and a high lymphocyte count (Figure 1A). No immature cells (blasts) suggestive of leukemia were observed. Flow cytometry of peripheral blood was performed and did not reveal an immunophenotypically abnormal population. Interestingly, approximately 80% of white blood cells were T cells, with a CD4/CD8 ratio of approximately 3.5:1 (Figure 1B–D). Testing for parvovirus and antineutrophil antibodies was negative. A respiratory pathogen panel performed by multiplex reverse transcriptase PCR amplification was positive for rhino/enterovirus. Given the leukopenia without evidence of leukemia, the neutropenia was initially thought to be due to viral suppression.

### 3.2. Persistence of Cytopenia and Bone Marrow Evaluation

As the patient’s blood counts did not recover spontaneously, a bone marrow procedure was performed. Flow cytometry of bone marrow aspirate revealed no immunophenotypic evidence of leukemia and, once again, a predominance of T lymphocytes (approximately 65% of total cells), with a CD4/CD8 ratio of approximately 3:1. Accordingly, the bone marrow aspirate showed mostly lymphocytes, with some histiocytes demonstrating evidence of hemophagocytosis (Figure 1E) and a decreased myeloid-to-erythroid ratio (M:E ratio: 0.8:1). The monocyte count was 4% by morphology and 3% of total cells by flow cytometry for the bone marrow aspirate. The natural killer (NK) cell count was 2.3% of total cells, as determined by flow cytometry. The bone marrow core biopsy was hypercellular, nearly 100% (Figure 1F), with increased histiocytes (Figure 1G) and lymphocytes. A mild increase in fibrosis (MF-1) was observed using a reticulin stain, which was performed to evaluate fibrosis in hypercellular marrow (Figure 1H). Stains for CD30, ALK1, and CD1a and Epstein–Barr encoding region (EBER) in situ hybridization were also performed during a bone marrow core biopsy to evaluate for lymphoid malignancies, Langerhans histiocytosis, and the possibility of EBV infection; all were negative.

Considering the cytopenia and hemophagocytosis noted in the patient’s bone marrow, an extensive work-up for HLH was initiated. Because HLH is commonly seen in the setting of viral infection in children, particularly EBV infections, testing for cytomegalovirus and Epstein–Barr virus was performed. Testing for CMV was negative, while testing for Epstein–Barr virus antibodies revealed positive IgG but negative IgM. Additional analysis revealed increased soluble CD25 (interleukin receptor 2; 56,663.3 pg/mL). According to the HLH-2004 diagnostic criteria, five of the following eight criteria must be met for diagnosis: fever, splenomegaly, cytopenia (at least bicytopenia, involving hemoglobin, platelets, and neutrophils), hypertriglyceridemia or hypofibrinogenemia, hemophagocytosis, hyperferritinemia, low or absent NK-cell activity, and elevated sCD25 levels [14]. In our patient, the observed findings included fever, cytopenia (anemia and neutropenia), hemophagocytosis in bone marrow, increased soluble CD25, and increased triglycerides. Splenomegaly was not present. However, ferritin level and NK cell activity were not determined; as these tests were ordered and were canceled due to the detection of the *KMT2A-MLLT3* fusion transcript by next-generation sequencing (NGS) shortly after the bone marrow procedure. Cytogenetic studies revealed abnormal mosaic female karyotype: 45,XX,t(9;11)(p21q23),der(14;15)(q10;q10)?c in seven metaphase cells and 45,XX,der(14;15)(q10;q10)?c in three metaphase cells. Considering the patient’s genetic findings indicative of leukemia, the absence of the typical signs of leukemia (no increase in blasts or immunophenotypically abnormal cells), a diagnosis of leukemia could not be made at this point. Therefore, the patient was closely followed.

### 3.3. Follow-Up of Evolving Leukemia to Diagnosis and Treatment

Three weeks after the bone marrow procedure, a peripheral blood smear was reviewed and no blasts were identified. In-house flow cytometry did not reveal an immunophenotypically abnormal population. The sample was sent to a reference lab to perform more sensitive testing by flow cytometry using a minimal residual disease (MRD) analysis, which was also negative. Interestingly, once more, the majority of the white blood cells were T lymphocytes (approximately 89%).

**Figure 2:**
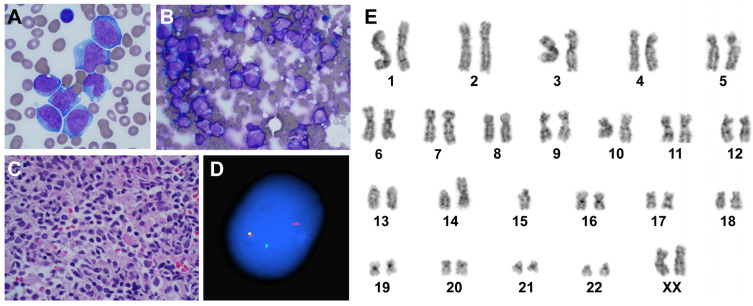
Peripheral blood and bone marrow with AML diagnosis. (**A**) Peripheral blood showing blasts that are large, with moderately abundant basophilic cytoplasm, some with oval and some with folded nuclei, finely dispersed chromatin, scattered fine azurophilic granules and cytoplasmic vacuoles (Wright–Giemsa stain, magnification ×1000). (**B**) Bone marrow aspirate and (**C**) bone marrow biopsy showing numerous blasts (Wright–Giemsa stain, magnification ×400). (**D**) Interphase FISH with *KMT2A (MLL)* rearrangement. (**E**) Standard G-banded karyotype of the bone marrow showing an abnormal female karyotype with t(9;11)(p21;q23) and a constitutional, balanced Robertsonian translocation der(14;15) (q10;q10)?c.

Four weeks after her last follow-up (seven weeks after her initial bone marrow procedure), a review of the peripheral blood smear revealed blasts that were large, with moderately abundant basophilic cytoplasm, some with oval and some with folded nuclei, and finely dispersed chromatin (Figure 2A). Flow cytometry performed on her peripheral blood and, later, bone marrow aspirate revealed 13.5% and 82% abnormal myeloblasts, respectively. Numerous blasts were seen on bone marrow aspirate (Figure 2B) as well as bone marrow biopsy (Figure 2C). The immunophenotype was consistent with a diagnosis of acute myeloid leukemia (AML)/acute monocytic leukemia, with myeloblasts expressing CD4, CD13 (dim), CD15 (variable), CD33 (bright), CD38, CD45, CD64, CD117 (dim), HLA-DR (bright); and negative for CD2, CD3, CD5, CD7, CD8, CD14, CD34, CD56, myeloperoxidase, and B cell markers. NGS of bone marrow aspirate detected *KMT2A::MLLT3* fusion (classified as Tier I—variant of strong clinical significance), as well as a pathogenic variant in the *PTPN11* (S502A) and *FLT3* (A680V), (both classified as Tier II—variants of potential clinical significance). Fluorescence in situ hybridization (FISH) for rearrangement of *KMT2A (MLL)* was positive (Figure 2D), and cytogenetic studies revealed abnormal female karyotype with 45,XX,add(7)(q32),t(9;11)(p21;q23),der(14;15)(q10;q10)?c in twenty metaphase cells (Figure 2E).

The patient underwent treatment per COG study AAML1831. The end-of-induction bone marrow was negative for AML minimal residual disease by flow cytometry. Notably, a bone marrow core biopsy showed 70–80% cellularity, with the focal hypercellular area (>95%) having an increase in CD68-positive monocytes/histiocytes. Considering that flow cytometry for AML MRD and *KMT2A* rearrangement by FISH were negative, these findings were thought to be the result of rebound hematopoiesis. Additionally, cytogenetic studies showed a normal female karyotype with a constitutional, balanced Robertsonian translocation. The patient remained in remission 6 months after the initial diagnosis.

## 4. Discussion

The presentation of HLH can be variable and nonspecific, and, in a young child, primary HLH due to genetic abnormality is more common than malignancy-associated HLH. Epstein–Barr virus (EBV) infection is a major contributing factor in both primary and malignancy-associated HLH. Studies show that nearly half of pediatric patients with malignancy-associated HLH also have EBV infection [9]. The malignancy-associated HLH in a young child is usually associated with lymphoid neoplasms, more commonly of T cell and NK cell lineages, and less commonly due to myeloid malignancies [7,8,9,15,16]. While myeloid malignancies are rarely seen in malignancy-associated HLH, they are primarily observed in the setting of immunosuppression (e.g., chemotherapy or stem cell transplant) rather than in patients whose HLH is triggered by AML itself [10,11]. Only a handful of pediatric patients with malignancy-triggered HLH caused by AML have been reported [9]. As in our patient, in one study, 2 out of 27 patients (14 years old and 1.9 years old) did not have a diagnosis of AML at the time of their initial HLH presentation [9]. The AML diagnosis was made after the first relapse of HLH, which was diagnosed 30 and 60 days after the initial HLH symptoms, respectively. Both patients exhibited hemophagocytosis in the bone marrow, but further details like blood morphology and immunophenotyping were not provided. The 14-year-old patient was EBV-positive, had chromosomal abnormalities (1q-, 12p+), and, unfortunately, died, whereas the 1.9-year-old patient was EBV-negative, had normal chromosomal analysis, and survived after treatment.

While the association between malignancy-triggered HLH and AML is not fully understood, the high mortality rate (52–64% survival) associated with malignancy-triggered HLH highlights the importance of early malignancy detection and treatment [7,8,9]. In this case, NGS and FISH analyses were crucial for diagnosis and guiding treatment management. Identification of the *KMT2A::MLLT3* fusion transcript indicated malignancy-associated HLH in the setting of evolving leukemia, even though the diagnosis of leukemia could not be made at that point. No increase in blasts or immunophenotypically abnormal cell population was identified by flow cytometry at presentation or even later, when performed by the reference lab utilizing MRD analysis. Therefore, the abnormal cell lineage was unknown, as was the type of leukemia that was evolving. A *KMT2A::MLLT3* fusion transcript has been reported in different types of leukemias: AML, acute lymphoblastic leukemia/lymphoma (ALL) of T- and B-cell lineage, as well as mixed-phenotype acute leukemia [17,18,19,20,21]. Furthermore, the diagnosis and classification of acute leukemias are based on the International Consensus Classification (ICC) and the World Health Organization 5th edition classification (WHO-HAEM5) [22,23]. According to the diagnostic criteria of both WHO-HAEM5 and ICC, a diagnosis of ALL, AML, or mixed-phenotype acute leukemia could not be made due to the absence of increased or immunophenotypically abnormal blasts. Applying the ICC diagnostic criteria for AML, this case could not be classified as AML, as 10% or more blasts are required for diagnosis. Although WHO-HAEM5 considers the presence of <20% blasts diagnostic for AML with *KMT2A* rearrangements, the absence of increased or abnormal blasts still precluded the AML diagnosis.

Acute leukemia with t(9;11)(p21.3;q23.3); *KMT2A::MLLT3* can occur at any age but is more common in children, particularly in the first two years of life. AML with t(9;11)(p21.3;q23.3) has an intermediate risk, which is superior to that of AML with other *KMT2A* (11q23.3) translocations [24]. The prognosis and risk in association with ALL might be different since the worst outcome was reported for the small number of patients with *KMT2A::MLLT3* T-ALL [25]. Although the *KMT2A::MLLT3* fusion transcript is more common in AML, particularly AML with monocytic features, and our patient had an increase in histiocytes/monocytes in bone marrow biopsy, these monocytes/histiocytes did not reveal an immunophenotypic abnormality. Furthermore, there is no definitive marker that can be used for the evaluation of immature monocytes (monoblasts and promonocytes). For example, CD34 is often used for the evaluation of the percentage of blasts in the bone marrow core biopsy of patients with AML; however, immature monocytes are frequently negative for CD34.

## 5. Conclusions

The presentation of evolving acute monocytic leukemia can be nonspecific, overlapping with other conditions, such as HLH-like manifestations, without an increase in immature cells or monocytes in the peripheral blood and bone marrow aspirate. Furthermore, although evaluation of the bone marrow core biopsy shows an increase in monocytes, there are no definitive markers that can be used to demonstrate immaturity or the clear abnormality of monocytes/histiocytes, thereby posing significant challenges for diagnosis. To date, there are only a few reports describing malignancy-associated HLH in pediatric patients, with very few patients having reported malignancy-triggered HLH in association with AML. While the data are limited, it should also be noted that the AML diagnosis might not be apparent at the initial HLH presentation. Therefore, maintaining a broad differential diagnosis for HLH and taking a comprehensive approach with NGS and/or cytogenetic testing may facilitate early diagnosis and appropriate treatment.

## Data Availability

The datasets used and/or analyzed during the current study are available from the corresponding author on reasonable request.

## Abbreviations

The following abbreviations are used in this manuscript:

HLH	Hemophagocytic lymphohistiocytosis
NGS	Next-generation sequencing
MRD	Minimal residual disease
AML	Acute myeloid leukemia
FISH	Fluorescence in situ hybridization
ALL	Acute lymphoblastic leukemia/lymphoma
ICC	International Consensus Classification ICC
